# Characterization of Perioperative Serotonin in Patients Undergoing Orthotopic Liver Transplantation

**DOI:** 10.3390/jcm13092640

**Published:** 2024-04-30

**Authors:** Tobias Zott, David Pereyra, Isabelle Kersten, Max Ortner, Maria Noelle Hüpper, Patrick Starlinger, Gabriela A. Berlakovich, Gerd R. Silberhumer

**Affiliations:** 1 Clinical Department of Transplantation, University Clinic for General Surgery, Medical University of Vienna, 1090 Vienna, Austriagerd.silberhumer@meduniwien.ac.at (G.R.S.); 2 Clinical Department of General Surgery, University Clinic for General Surgery, Medical University of Vienna, 1090 Vienna, Austria; 3 Department of General Surgery, LMU Munich, 81377 Munich, Germany; 4 Department of General Surgery, Mayo Clinic, Rochester, MN 55902, USA

**Keywords:** liver transplantation, liver regeneration, serotonin

## Abstract

**Background:** Platelets were shown to be relevant for liver regeneration. In particular, platelet-stored serotonin (5-HT) proved to be a pro-regenerative factor in this process. The present study aimed to investigate the perioperative course of 5-HT and evaluate associations with patient and graft outcomes after othotopic liver transplantation (OLT). **Methods:** 5-HT was quantified in plasma and serum of 44 OLT recipients perioperatively, and in their respective donors. Olthoff’s criteria for early allograft dysfunction (EAD) were used to evaluate postoperative outcomes. **Results:** Patients with higher donor intra-platelet 5-HT per platelet (IP 5-HT PP) values had significantly lower postoperative transaminases (ASAT POD1: *p* = 0.006, ASAT POD5: *p* = 0.006, ASAT POD10: *p* = 0.02, ALAT POD1: *p* = 0.034, ALAT POD5: *p* = 0.017, ALAT POD10: *p* = 0.04). No significant differences were seen between postoperative 5-HT values and the occurrence of EAD. A tendency was measured that donor IP 5-HT PP is lower in donor-recipient pairs that developed EAD (*p* = 0.07). **Conclusions:** Donor IP 5-HT PP might be linked to the postoperative development of EAD after OLT, as higher donor levels are correlated with a more favorable postoperative course of transaminases. Further studies with larger cohorts are needed to validate these findings.

## 1. Introduction

Orthotopic liver transplantation remains the only potentially curative method to treat a variety of end-stage liver diseases while extending life expectancy in this patient cohort [1,2]. However, postoperative complications frequently occur and a mortality rate of 11% within the first six months after transplantation is reported for patients undergoing OLT [3,4]. Specifically, early allograft dysfunction (EAD) as a non-surgical complication occurs in 15–30% of all patients after OLT [5]. Yet, the pathophysiology of EAD is not completely understood at this point, which renders EAD an irreversible complication that crucially affects post-transplant morbidity and survival [5,6].

In this context, platelets and platelet-stored molecules were recently reported to be involved in the process of liver regeneration after partial liver resection (LR) [7]. Impaired platelet function and compromised platelet degranulation were associated with the development of post-hepatectomy liver failure after LR [7,8,9]. Particularly, platelet-stored 5-HT was investigated as a central pro-regenerative molecule in rodents as well as in humans [10,11,12]. In regards to OLT, thrombopenia was linked to deteriorated graft function and reduced short- and long-term patient survival [12,13]. Still, the relevance of 5-HT for patient outcomes after OLT has not been explored so far.

The aim of this study was to monitor the perioperative course of 5-HT in patients undergoing OLT and to evaluate potential associations with adverse outcomes as well as the development of EAD.

## 2. Materials and Methods

### 2.1. Study Population

Forty-four patients undergoing OLT at the Medical University of Vienna between 2018 and 2021 were included in this study. The study was approved by the ethics committee of the Medical University of Vienna and conducted in accordance with both the declarations of Helsinki and Istanbul. Informed consent of the recipients was obtained before sampling. Only grafts of donors after brain death (DBD) were considered as the study population. Of note, patients receiving platelet transfusions were excluded from this study. Data on routine blood parameters of donors and correlating recipients were documented in a prospectively maintained database. For each donor, the donor risk index (DRI) was calculated as previously published [14]. 

### 2.2. Material Acquisition

**Figure 1:**
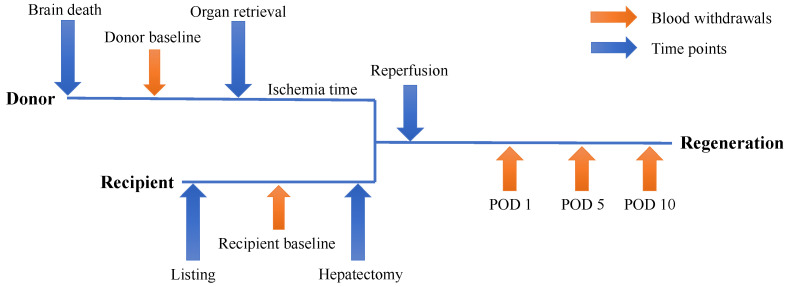
Timeline of blood sample acquisition. Two preoperative (donor and recipient) and three postoperative blood withdrawals on day one (POD 1), five (POD 5) and ten (POD 10).

To evaluate perioperative platelet activation and IP factors of liver regeneration, plasma, serum and platelet extracts of recipients undergoing liver transplantation were collected prior to liver transplantation (4–12 h before transplant surgery started), 30 min after reperfusion of the transplanted graft, 24 h after liver transplantation, on the 5th and 10th postoperative day (POD). To accurately evaluate liver regeneration, regular blood parameters including blood counts and coagulation tests were performed at the same time points (routine hospital procedure). Patients were evaluated prospectively for perioperative liver dysfunction, morbidity and mortality. Additionally, plasma, serum and platelet extracts were collected from the liver donors immediately prior to the organ retrieval procedure. The timetable is illustrated in Figure 1.

### 2.3. Plasma and Serum Preparation

Optimized plasma and serum sample preparation was carried out as previously described [11]. Measurements of 5-HT values were carried out using enzyme immunoassay for the quantitative determination of 5-HT in human serum and plasma (RE59121) by IBL International GmbH. Subsequently, IP 5-HT was calculated by subtracting plasma from serum 5-HT.

### 2.4. Definition of Outcome

EAD was defined and evaluated according to the criteria described by Olthoff et al. [15]. Briefly, one of the following criteria had to be present to fulfill Olthoff’s criteria for EAD: bilirubin ≥ 10 mg/dL on POD7, international normalized ratio (INR) ≥ 1.6 on POD7, alanine or aspartate aminotransferases (ALAT/ASAT) > 2000 IU/L within the first seven PODs. Patients fulfilling any of these criteria were referred to as “EAD”, all others were defined as “no EAD”. 

### 2.5. Statistical Analyses

Statistics were carried out using SPSS Statistics Version 27.0.1.0 (IBM International, Armonk, NY, USA) and were based on non-parametric testing. To determine differences in 5-HT values or other blood parameters between outcome cohorts (i.e., EAD vs. no EAD), Mann–Whitney U tests were used, while differences between time points were evaluated via Wilcoxon signed rank tests. Boxplot illustrations were given without outliers and extreme values to improve resolution. Bivariate correlations (Spearman’s rho) were used to test for associations between two metric variables and scatter plots were used for visualization of these results.

## 3. Results

### 3.1. Patient Demographics

Characteristics of the study cohort can be found in Table 1. In addition, respective donor samples were obtained and donor characteristics are visualized in Table 2. The main listing diagnoses were alcoholic liver cirrhosis (ALCI; *n* = 17, 38.6%) followed by hepatocellular carcinoma (HCC; *n* = 6, 13.6%) and primary sclerosing cholangitis (PSC; *n* = 5, 11.4%). Nine patients (20.5%) met the criteria for EAD. Patients who developed EAD had significantly longer hospital stays (*p* = 0.027). The median follow-up period in this cohort was 734.5 days. Two recipients died during follow-up (4.5%).

All of the donors in this study were DBD donors. The most common causes of death (75%) were cerebral pathologies. Donors of recipients who later developed EAD had significantly higher lactate dehydrogenase (LDH, *p* = 0.027). No significant difference in DRI was seen between donors of recipients who later developed EAD and those who did not. 

### 3.2. Serum and Intra-Platelet 5-HT Decrease after Liver Transplantation with a Concomitant Rise of 5-HT in Plasma

**Figure 2:**
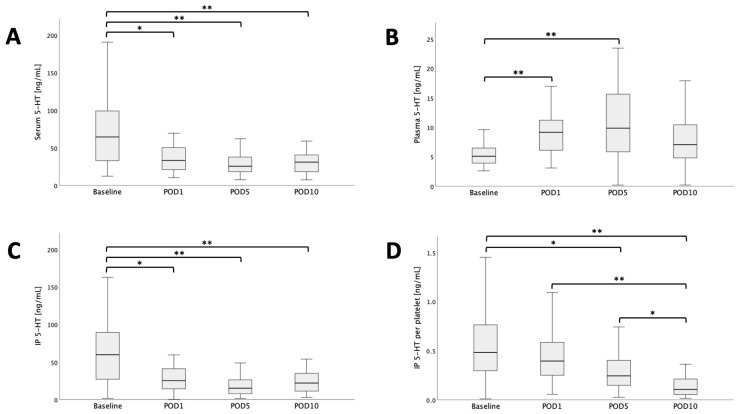
Perioperative course of serum- (**A**), plasma- (**B**) and IP 5-HT (**C**) as well as IP 5-HT PP (**D**). Significant differences are highlighted. * *p* < 0.05. ** *p* < 0.005.

The course of perioperative 5-HT was analyzed and an immediate decrease in serum 5-HT on POD1 was observed when compared to pre-transplant levels (Table 3; median serum 5-HT baseline = 64.8 ng/mL, median serum 5-HT POD1 = 33.4 ng/mL; *p* = 0.032; Figure 2A). This decrease persisted until POD5 (median serum 5-HT POD5 = 25.7 ng/mL, median serum 5-HT POD10 = 31.1 ng/mL; baseline vs. POD5 *p* < 0.001, baseline vs. POD10 *p* = 0.001; Figure 2A). Of note, the decrease in serum 5-HT was paralleled by a sudden increase in plasma 5-HT on POD1 and POD5 when compared to baseline plasma 5-HT levels (median plasma 5-HT baseline = 5.1 ng/mL, median plasma 5-HT POD1 = 9.2 ng/mL, median plasma 5-HT POD5 = 9.9 ng/mL; baseline vs. POD1 *p* = 0.003, baseline vs. POD5 *p* < 0.001, Figure 2B). In regards to intra-platelet (IP) 5-HT, the dynamic was comparable to serum 5-HT with a persistent decrease until POD5 (median IP 5-HT baseline 59.9 ng/mL, median IP 5-HT POD1 25.3 ng/mL, median IP 5-HT POD5 15.1 ng/mL, median IP 5-HT POD10 21.9 ng/mL; baseline vs. POD1 *p* = 0.011, baseline vs. POD5 *p* < 0.001, Figure 2C). However, when normalizing IP 5-HT to the evaluated platelet count and thereby calculating per platelet 5-HT content, a continuous postoperative decrease was observed (median IP 5-HT PP baseline 0.49 ng/mL, median IP 5-HT PP POD1 0.39 ng/mL, median IP 5-HT PP POD5 0.25 ng/mL, median IP 5-HT PP POD10 0.11 ng/mL; baseline vs. POD5 *p* = 0.005, baseline vs. POD10 *p* < 0.001, Figure 2D). 

### 3.3. Recipients 5-HT Dynamics Do Not Differ between Recipients with or without Early Allograft Dysfunction

**Figure 3:**
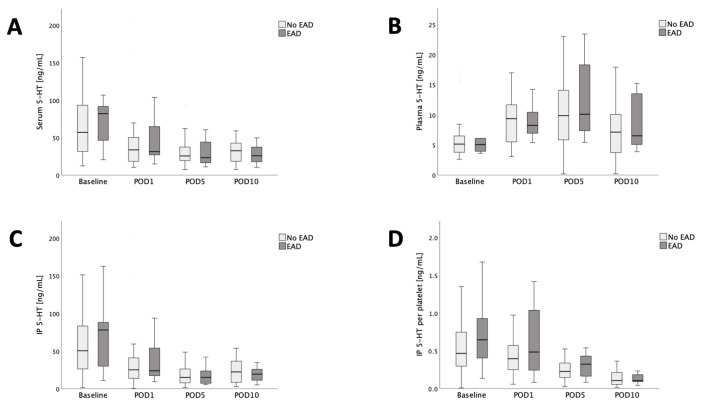
No significant differences in the recipients’ perioperative course of serum- (**A**), plasma- (**B**) and IP 5-HT (**C**) as well as IP 5-HT PP (**D**), each dichotomized by EAD.

As 5-HT might play a role in the development of EAD, differences in 5-HT concentrations between patients with or without EAD were investigated next. No significant differences in serum or plasma 5-HT, and IP 5-HT or IP 5-HT per platelet levels could be shown between recipients developing or not developing EAD (Figure 3). Notably, while there were no differences in 5-HT regardless of the evaluated compartment, patients who met EAD criteria had significantly lower platelet counts on POD10 (median platelet count no EAD = 196 × 10^9^/L, median platelet count EAD = 123 × 10^9^/L; *p* = 0.026).

### 3.4. Donor IP 5-HT per Platelet Might Be Associated with Development of Early Allograft Dysfunction

**Figure 4:**
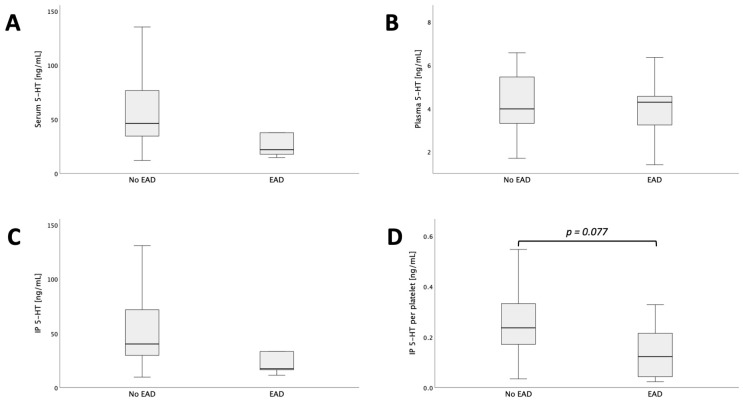
Donor values of serum- (**A**), plasma- (**B**) and IP 5-HT (**C**) as well as IP 5-HT PP (**D**), each dichotomized by EAD. Moreover, the tendency of lower donor IP 5-HT PP in patients that later develop EAD can be seen in Subfigure D.

Next, we aimed to evaluate whether donor 5-HT might be associated with the development of EAD in respective recipients. There was no significant difference in serum and plasma 5-HT between patients with or without EAD development (donor serum 5-HT: *p* = 0.170, donor plasma 5-HT: *p* = 0.766; Figure 4A,B). Similarly, there was no significant difference in donor IP 5-HT between those cohorts (donor IP 5-HT: *p* = 0.185, Figure 4C). However, after normalization to platelet counts, a tendency towards lower donor IP 5-HT PP was observed in donor-recipient pairs that postoperatively developed EAD (donor IP 5-HT PP: *p* = 0.077, Figure 4D).

### 3.5. Low Donor IP 5-HT PP Is Associated with Elevated Transaminases after Liver Transplantation

**Figure 5:**
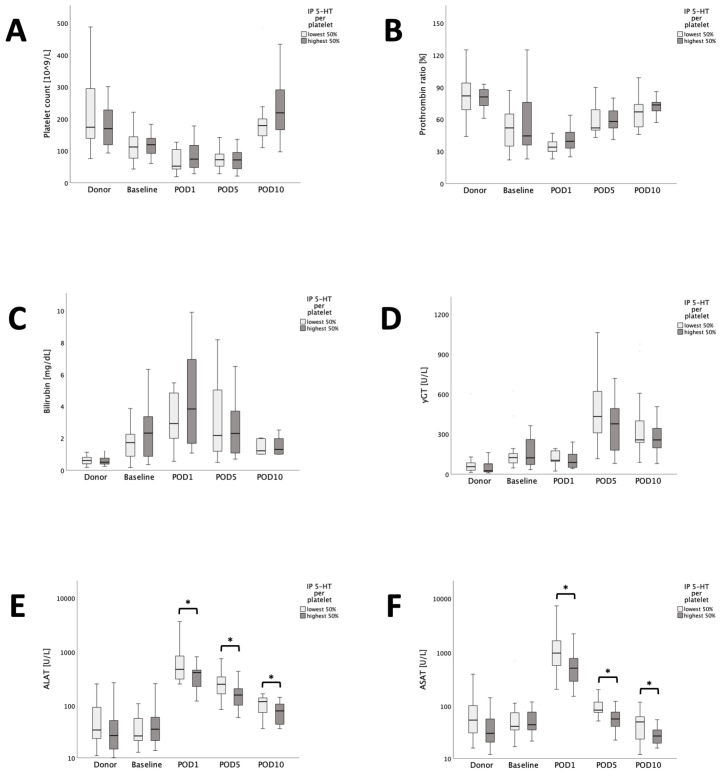
Comparison of liver parameters with our cohort dichotomized by median donor IP 5-HT PP. Platelet count (**A**), prothrombin ratio (**B**), bilirubin (**C**), GGT (**D**), ALAT (**E**) and ASAT (**F**) are shown. The significant differences in ASAT and ALAT are highlighted. * *p* < 0.05.

Ultimately, the aim was to further explore the postoperative dynamics in circulating parameters of liver damage and function between donor-recipient pairs with low or high IP 5-HT PP donors. For this, the cohort was divided into two subgroups according to the median of this parameter and liver-specific blood parameters were compared between the two subgroups, as visualized in Figure 5. There was no difference in perioperative platelet count, prothrombin time, bilirubin and gamma-glutamyl transferase (GGT) between the evaluated subgroups. Likewise, baseline ASAT and ALAT were comparable between patients with high or low IP 5-HT PP. However, postoperatively ASAT and ALAT were significantly increased in the subgroup characterized by low donor IP 5-HT PP values (ASAT POD1: *p* = 0.006, ASAT POD5: *p* = 0.006, ASAT POD10: *p* = 0.02; ALAT POD1: *p* = 0.034, ALAT POD5: *p* = 0.017, ALAT POD10: *p* = 0.04). 

**Figure 6:**
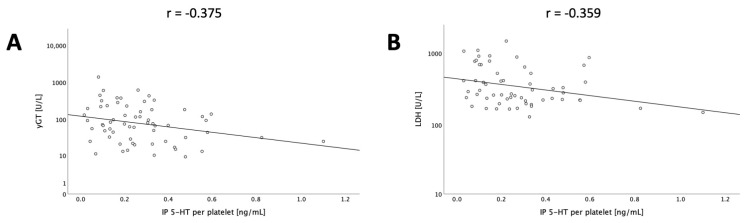
Scatter plots of donor IP 5-HT PP and donor values of GGT (**A**) and LDH (**B**) with.

In order to explore potential underlying reasons for this observation, we evaluated correlations of donor IP 5-HT PP and liver function tests in donors and observed a negative correlation of this parameter with GGT (*n* = 35, r = −0.375, *p* = 0.27) and LDH (*n* = 34, r = −0.359, *p* = 0.37), as shown in Figure 6, potentially indicating improved organ quality in donors with high IP 5-HT PP.

## 4. Discussion

In this study, we evaluated the association of IP 5-HT PP of donors for OLT and the outcome of correlating recipients in terms of EAD development. We observed a tendency towards decreased IP 5-HT PP levels in donors later resulting in EAD in the respective recipients. Further subgroup analyses by grouping into 50% highest and 50% lowest donor IP 5-HT PP values revealed significantly lower courses of postoperative levels of ASAT and ALAT in the 50% highest donor group. By that, we provide exploratory evidence that donor IP 5-HT PP may be connected to the occurrence of EAD and might potentially be associated with the dynamics of postoperative graft function. Additionally, we observed a negative correlation between donor IP 5-HT PP and LDH as well as GGT in donors for OLT, which further suggests that donor IP 5-HT PP might be connected to organ quality and hence influence EAD development after OLT.

Platelets and 5-HT were described as essential modulators of liver regeneration after surgical resection [16]. Alkozai et al. found immediate postoperative low platelet count as an independent risk factor for delayed postoperative recovery of liver function after LR [17]. In mouse models, Lesurtel et al. showed that impaired platelet activity leads to failure of cellular proliferation after partial hepatectomy, which was pronounced in mice lacking 5-HT indicating a specific relevance of this molecule during liver regeneration [16]. Interestingly, the expression of 5-HT receptors in the liver is increased after LR. Further, antagonists to these receptors or the elimination of the limiting enzyme in 5-HT synthesis disrupted liver regeneration in knock-out mice [16]. In addition, it was demonstrated that IP 5-HT was of major clinical relevance in predicting post-hepatectomy liver failure and morbidity in the setting of LR [11]. 

Due to our knowledge, limited data are available in the setting of OLT. Yoshizumi et al. investigated the course of 5-HT in 34 healthy living-donor hepatectomies [18]. Here, serum 5-HT levels and platelet counts decreased until POD3 after surgery and increased thereafter. Han et al. monitored the course of 5-HT in 32 recipients undergoing living-donor liver transplantation until five hours after reperfusion [19]. We were able to follow the trend of declining serum- and IP 5-HT until POD5 followed by an increase towards POD10, while plasma 5-HT levels increased towards POD5 and dropped towards POD10. Interestingly, IP 5-HT PP continuously decreased towards POD10. While we were unable to observe an association between perioperative 5-HT and patient outcome after OLT, the present investigation suggests a potential relevance of donor IP 5-HT PP for patient outcome. Indeed, liver regeneration in the setting of OLT was previously shown to be affected by donor-specific parameters [20]. Several donor risk factors for EAD have been identified and resulted in the establishment of different donor risk indices to evaluate organ quality [14,21,22,23]. Nevertheless, in our study population, DRI did not show significant differences regarding EAD. 

During OLT, platelets and leukocytes synergistically trigger sinusoidal endothelial cell apoptosis upon reperfusion of static cold storage preserved organs. In the normothermic hepatic ischemia mouse model platelet-derived 5-HT was a mediator of hepatocyte proliferation in the postischemic liver [24]. Accordingly, donor IP 5-HT PP might be involved in the preservation of liver grafts from ischemia-reperfusion injury. In fact, we observe increased transaminases in the postoperative course for patients who received a liver graft from low IP 5-HT PP donors as a potential consequence of more pronounced ischemia-reperfusion injury in respective patients.

While these pathomechanistic associations need to be investigated in more detail, the observations made in this study state the role of donor IP 5-HT PP as an indicator or biomarker for outcome after OLT. In fact, biomarkers for prediction of EAD development are scarce, yet, relevant in order to improve post-transplant outcome. While the field of OLT surgery has seen vast improvements over the last decades and one-year survival after OLT reached 80% in the Eurotransplant region between 2007 and 2014 [25], postoperative EAD development and associated morbidity and mortality, still represent major clinical concerns [26,27]. In this regard, donor IP 5-HT PP could serve as a potential predictor for EAD development that is entirely independent of recipient risk parameters. However, it needs to be mentioned that the evaluation of IP 5-HT is prone to artifacts due to in vitro platelet activation and potential inhibition [28,29]. 

We observed vast differences in 5-HT concentrations between our study population and previously published investigations, which further also impacted the observed perioperative dynamics. Our cohort displays a median baseline serum 5-HT value of 64.8 ng/mL, while the baseline serum 5-HT value in the cohort of Han et al. was substantially lower with a median of 24.5 ng/mL. This might be related to the fact that in our study a large part of patients suffered from end-stage liver cirrhosis. In regards to dynamics of serum 5-HT, Han’s group measured a decrease in the anhepatic phase, an increase during the first hour after reperfusion and a continuous decrease thereafter until five hours postoperatively [19]. We were able to follow this decrease until POD5 in the present analysis. Serum 5-HT represents the 5-HT pool after platelet activation. The immediate postoperative decrease might be caused by perioperative platelet activation as well as hemodilution. In contrast, the median baseline plasma value of 5.1 ng/mL increased significantly to 9.2 ng/mL on POD1 (*p* = 0.003), which is suggestive of an effect dependent on perioperative platelet activation. After another slight increase toward POD5, plasma 5-HT then tended to decrease toward POD10. Noticeably, IP values of 5-HT were similar to serum levels, as most 5-HT in circulation stems from platelets. IP 5-HT PP started at a baseline of 0.49 ng/mL and continuously decreased postoperatively. Between POD5 and POD10, IP 5-HT PP decreased significantly to 0.11 ng/mL (*p* = 0.014). This could be due to platelet counts regenerating faster than the 5-HT pool. Regarding platelet counts, our cohort started with a median baseline value of 113 × 10^9^/L, while Han et al. measured a baseline value of 54 × 10^9^/L, which might further explain the differences in 5-HT concentrations that were evaluated. Yet, a standardized protocol for preanalytical blood workup and evaluation of IP 5-HT is necessary in order to allow for the broad application of this parameter as a biomarker.

Limitations of this study are the small number of included patients, which is owed to the enormous logistic efforts to collect the study material. Further, we assume that a procedure such as organ retrieval with consecutive transplantation in severely ill patients is associated with several side risk factors not directly related to organ quality. Analyzed donors represent a positive selection of potential liver donors as these organs were finally used for transplantation. It has to be mentioned that around 60% of grafts were preconditioned by perfusion systems, the impact of this is subject of ongoing research.

In conclusion, we found a potential association of donor IP 5-HT PP and post-transplant outcome in terms of EAD. IP 5-HT PP might be a relevant modulator of EAD development after OLT. However, further investigations regarding the pathophysiological involvement of 5-HT during OLT and EAD development are needed. Yet, donor IP 5-HT PP could serve as a potential biomarker for the prediction of EAD, which is entirely independent of donor risk factors. Ultimately, the use of this potential biomarker in association with commonly applied parameters for the evaluation of organ quality and recipient risk might improve the overall outcome of patients subjected to OLT.

## Figures and Tables

**Table 1:** Recipient demographics.

Study Cohort	Entire Cohort (*n* = 44)	EAD (*n* = 9)	No EAD (*n* = 35)	*p*
Parameter	N (%), Median (IQR)			
Sex				1.000
Male	33 (75%)	7 (77.8%)	26 (74.3%)	
Female	11 (25%)	2 (22.2%)	9 (25.7%)	
Age at OLT [a]	55.4 (49.1–63.4)	50.8 (43.0–62.1)	57.9 (50.2–64.1)	0.300
BMI at OLT [kg/m^2^]	26.4 (22.3–32.0)	27.7 (24.3–33.5)	26.3 (21.7–31.8)	0.274
MELD at OLT	16.2 (10.9–20.9)	13.2 (8.6–20.6)	16.4 (11.6–21.3)	0.274
Listing diagnosis				
ALCI	17 (38.6%)	1 (11.1%)	16 (45.7%)	0.060
HCC	6 (13.6%)	1 (11.1%)	5 (14.3%)	0.644
PSC	5 (11.4%)	1 (11.1%)	4 (11.4%)	0.733
Hepatitis	4 (9.1%)	2 (22.2%)	2 (5.7%)	0.180
Benign liver tumors	3 (6.8%)	1 (11.1%)	2 (5.7%)	0.506
Other	9 (20.5%)	3 (33.4%)	6 (17.1%)	0.260
Surgical revision				0.101
No	34 (77.3%)	5 (55.6%)	29 (82.9%)	
Yes	10 (22.7%)	4 (44.4%)	6 (17.1%)	
Vascular complications	2 (20%)	1 (25%)	1 (16.7%)	
Biliary complications	4 (40%)	1 (25%)	3 (50%)	
Bleeding/Hematoma	4 (40%)	2 (50%)	2 (33.3%)	
Perfusion				0.925
No machine perfusion	17 (38.6%)	3 (33.3%)	14 (40%)	
Hypothermic	23 (52.3%)	5 (55.6%)	18 (51.4%)	
Normothermic	4 (9.1%)	1 (11.1%)	3 (8.6%)	
ICU stay [d]	7 (4–12)	12 (5–18)	6 (4–9)	0.125
Hospital stay [d]	20 (15–25)	24 (22–42)	18 (15–24)	0.027 *
Follow-up period [d]	734.5 (372.8–1088.5)	117 (55–673)	240 (44–500)	0.954
Death within follow-up period				0.371
No	42 (95.5%)	8 (88.9%)	34 (97.1%)	
Yes	2 (4.5%)	1 (11.1%)	1 (2.9%)	

* *p* < 0.05.

**Table 2:** Donor demographics.

Study Cohort	Entire Cohort (*n* = 44)	EAD (*n* = 9)	No EAD (*n* = 35)	*p*
Parameter	N (%), Median (IQR)			
Sex				0.431
Male	22 (61.1%)	3 (50%)	19 (63.3%)	
Female	14 (38.9%)	3 (50%)	11 (36.7%)	
Age [a]	55.5 (45.0–60.0)	53.5 (26.0–59.0)	56.5 (45.0–60.3)	0.548
BMI [kg/m^2^]	25.0 (22.3–29.0)	26.0 (21.8–31.2)	25.0 (22.8–29.0)	0.725
Cause of death				0.858
Cerebral	27 (75%)	4 (66.7%)	23 (76.7%)	
Trauma	5 (13.9%)	1 (16.7%)	4 (13.3%)	
Cardiovascular	4 (11.1%)	1 (16.7%)	3 (10%)	
Respiratory	0 (0%)	0 (0%)	0 (0%)	
Cardiac arrest				0.447
Yes	11 (35.5%)	2 (50%)	9 (33.3%)	
No	20 (64.5%)	2 (50%)	18 (66.7%)	
Alcohol abuse				0.715
Yes	6 (21.4%)	1 (20%)	5 (21.7%)	
No	22 (78.6%)	4 (80%)	18 (78.3%)	
Smoking				0.455
Yes	11 (40.7%)	1 (25%)	10 (43.5%)	
No	16 (59.3%)	3 (75%)	13 (56.5%)	
Diabetes				0.762
Yes	2 (6.3%)	0 (0%)	2 (7.1%)	
No	30 (93.8%)	4 (100%)	26 (92.9%)	
Hypertension				0.530
Yes	16 (48.5%)	2 (40%)	14 (50%)	
No	17 (51.5%)	3 (60%)	14 (50%)	
Laboratory				
AST	46 (24–83)	63 (47–220)	39 (22–77)	0.133
ALT	31 (15–75)	41 (15–118)	31 (15–75)	0.885
LDH	240 (192–330)	369 (266–1172)	234 (176–306)	0.027 *
GGT	39 (21–82)	60 (31–231)	31 (19–79)	0.250
Platelet count	173 (134–265)	283 (175–427)	170 (130–232)	0.135
DRI	1.56 (1.33–1.89)	1.57 (1.25–2.03)	1.56 (1.29–1.89)	0.881

* *p* < 0.05.

**Table 3:** Course of 5-HT.

Median (IQR) n	Baseline	POD1	POD5	POD10
Serum 5-HT [ng/mL]	64.8 (31.7–103.1) *n* = 44	33.4 (19.0–51.2) *n* = 43	25.7 (17.6–38.5) *n* = 43	31.1 (18.1–41.2) *n* = 42
Plasma 5-HT [ng/mL]	5.1 (3.9–6.5) *n* = 44	9.2 (5.8–11.5) *n* = 43	9.9 (5.8–16.7) *n* = 43	7.1 (4.6–10.6) *n* = 42
IP 5-HT [ng/mL]	59.9 (25.7–90.4) *n* = 44	25.3 (14.2–41.7) *n* = 43	15.1 (7.5–26.6) *n* = 43	21.9 (10.5–34.9) *n* = 42
IP 5-HT PP [ng/mL]	0.49 (0.29–0.77) *n* = 44	0.39 (0.23–0.59) *n* = 43	0.25 (0.15–0.41) *n* = 43	0.11 (0.05–0.28) *n* = 42

## Data Availability

The data that support the findings of this study are available from the corresponding author, upon reasonable request.
